# ‘It’s hard not to feel like somehow I fell short’: A discourse analysis of palliative care providers’ experiences with patients’ nonphysical suffering

**DOI:** 10.1177/26323524251379910

**Published:** 2025-09-30

**Authors:** Maxxine Rattner, Cheryl-Anne Cait

**Affiliations:** 1 School of Social Work, York University, Toronto, ON, Canada; 2 Faculty of Social Work, Wilfrid Laurier University, Kitchener, ON, Canada

**Keywords:** suffering, palliative care, nonphysical, existential, psychosocial, discourse analysis

## Abstract

**Background:**

The relief of patients’ suffering is central to the philosophy and practice of palliative care. Few studies have focused on interdisciplinary palliative care providers’ experiences in addressing patients’ nonphysical suffering—suffering that is emotional, psychological, social, spiritual, and/or existential in nature. This study contributes to and expands the empirical knowledge base in this area.

**Objective:**

To identify existing discourses within how palliative care providers talk about their experiences working with patients’ nonphysical suffering, and how these discourses may affect palliative care providers and impact clinical care.

**Design:**

This study’s methodological approach was informed by discourse analysis, grounded in a poststructural theoretical framework. Discourse analysis guided by poststructural theory highlights that: discourses are constructed; discourses are imbued with tensions and/or contradictions; and discourses *do* things—they have *effects*.

**Methods:**

Twenty-four interdisciplinary palliative care providers across Canada participated in semi-structured phone interviews in 2020. Eligible participants had a minimum of 2 years’ experience providing palliative care to adult patients in diverse settings, and self-identified as having experience working with patients’ nonphysical suffering.

**Results:**

Two competing discourses shape providers’ work with patients’ nonphysical suffering: (1) ‘Expectations of palliative care to relieve nonphysical suffering’ and (2) ‘Barriers to nonphysical suffering’s relief’. The expectation that palliative care will relieve patients’ nonphysical suffering emanates from patients and families, from medical teams outside of palliative care, and from providers themselves. Multiple barriers to nonphysical suffering’s relief exist at the patient, provider, and systems levels. Providers may experience helplessness, and inadequacy, and can avoid patients’ nonphysical suffering when they feel unable to relieve it.

**Conclusion:**

Palliative care has long ascribed nonphysical suffering’s relief to the ability of a patient to transcend their own suffering, or a palliative care provider to facilitate/support that transcendence. Findings call into question and expand this long-standing discourse by pushing the field to consider how multiple intersecting barriers at patient, provider, and systems levels shape—and limit—palliative care’s call for the relief of nonphysical suffering. Findings have implications for patient care, and interdisciplinary palliative care training, education, and research.

## Introduction

The prevention and relief of suffering, both physical and nonphysical, remain central aims of palliative care.^1,2^ While ‘nonphysical suffering’ is a term that has been used in palliative care research,^3,4^ its use in practice is more limited. ‘Existential suffering’ is a more common focus in palliative care practice and research, though definitions and understandings remain contested.^3,5–12^ This study adopts the term ‘nonphysical suffering’ to refer to forms of suffering that are emotional, psychological, social, spiritual, and/or existential in nature.^13^ As these forms of suffering can overlap, ‘nonphysical suffering’ offers practitioners and researchers a way to acknowledge the whole of a patient’s suffering that lies beyond the physical domain. It is distinct from Dame Cicely Saunders’ important multi-dimensional conceptualization of ‘total pain’, which always includes a physical element alongside the nonphysical^14,15^; by contrast, nonphysical suffering does not necessarily have a physical source or manifestation. Research has shown that patients distinguish their experiences of physical suffering from nonphysical suffering,^16,17^ and that palliative care providers struggle more, and differently, with patients’ nonphysical suffering than with their physical suffering.^13,18,19^ The need to better understand how palliative care providers describe their experiences and challenges in working with patients’ nonphysical suffering, including the impact on themselves and the care they provide, was a key rationale for this study.

This study also intentionally uses the word ‘suffering’ rather than ‘distress’ or ‘pain’. While these terms are often used interchangeably in palliative care literature and practice,^19,20–23^, they are conceptually distinct: individuals may experience pain without suffering, and conversely, suffering without pain.^22,24–26^ Suffering is often understood to be more pervasive, enduring, and intense than distress,^20,21,26–28^ which is typically characterized as more transient or fleeting.^26^ A recent scoping review emphasized the significance of language in distinguishing between suffering, distress, and pain—particularly when such distinctions influence how discourse shapes both practice and research.^13^

Diverse research exists offering insights into nonphysical suffering,^7,29,30^ though few studies have centered interdisciplinary palliative care providers’ experiences specific to their work with patients’ nonphysical suffering: existing studies have either focused on providers’, patients’, and family members’ experiences with patients’ existential suffering specifically^3^; on nurses’ experiences working with ‘unrelieved’ suffering^19^; or interdisciplinary providers’ experiences with both physical and nonphysical suffering.^31–33^ A 2017 pilot qualitative study of providers working in diverse palliative care settings in an urban Ontario city was the first to explore interdisciplinary providers’ experiences working with patients’ nonphysical suffering, specifically. It revealed that providers lack training and education specific to working with patients’ nonphysical suffering; that both systemic barriers and the ‘intangibility’ of nonphysical suffering make it difficult to support; and that palliative care providers experience helplessness when they cannot relieve it.^34^ The study outlined in this paper was informed by and builds upon that pilot study and is the first Canada-wide study examining interdisciplinary palliative care providers’ experiences working with patients’ nonphysical suffering from a discursive lens. The study’s poststructuralist theoretical framework and discourse analysis methodology aim to increase awareness of discourses and how they shape practice, as well as how providers navigate tensions in their work with patients’ nonphysical suffering.

## Methodological and theoretical framework

Discourse analysis is a qualitative, interpretive methodology that can be flexibly adapted to a researcher’s needs and aims^35^; it has been used to study a wide range of topics in palliative care.^36–47^ Poststructuralism is the theoretical framework guiding this study. It seeks to make visible what may be less visible and to question taken-for-granted ideas and knowledge that shape practice across diverse fields.^48–52^ Poststructural understandings of ‘discourse’ guided this study: ‘discourse’ is defined as ‘the language practices through which knowledge, truth, our sense of selves and social relations are constructed. . .[and] through which we understand “reality” and act on it’,^53 (p.211)^ and as ‘structures of knowledge that influence systems of practices’.^54(p.57)^ The poststructural theoretical tenets guiding this study’s approach to discourse analysis are: (1) discourses are constructed; (2) discourses are imbued with tensions and/or contradictions; and (3) discourses *do* things; they have *effects* (please see ‘Data analysis’ below).^48,50,51^ From a poststructural perspective, ideas/knowledge can become so entrenched that they come to dominate ways of thinking and doing, often without questioning. For example, a long-standing—or dominant—discourse within palliative care is that patients can transcend their nonphysical suffering,^15,55–65^ and palliative care providers can facilitate this transcendence.^9,57,59,63–65^ Are such discourses present in how palliative care providers describe their experiences working with patients’ nonphysical suffering? And/or what other discourses, if any, may be present? The research question guiding this study was: What discourses are present in how palliative care providers describe their experiences with patients’ nonphysical suffering? A subsequent research question guiding this study was: Are there discursive *effects*? That is, what is the effect of identified discourses on palliative care providers and/or clinical care? This study’s theoretical underpinnings in poststructuralism allowed for these questions to be considered, shaping the study’s aims and analysis.

### Sampling

Purposive and snowball sampling were used; M.R. shared the recruitment poster via email with a select group of palliative care contacts across Canada, inviting them to circulate it within their networks. Participants were not previously known to the research team. Inclusion criteria were English-speaking providers, with a minimum of 2 years’ experience providing palliative care to adult patients in diverse settings (e.g., home care, inpatient palliative care unit, residential hospice, long-term care), who self-identified as having encountered, and/or worked with patients’ nonphysical suffering. A semi-structured interview guide was pilot-tested with two palliative care providers known to M.R. (one physician, one allied health professional). Participants were asked to reflect on and share experiences with patients’ nonphysical suffering from early in their career as well as more recently, including how, if at all, their approach to addressing nonphysical suffering has changed over the years. Following written informed consent, between July and September 2020, 24 palliative care providers from across Canada participated in this study exploring their experiences working with patients’ nonphysical suffering. Participants included social workers, music therapists, nurses, physicians, and an occupational therapist (see Table 1). The majority of participants identified as white, female, spiritual, and had 10–19 years of clinical experience working with adult patients in diverse palliative care settings (e.g., outpatient clinic, residential hospice, home care, hospital (palliative care consult team and inpatient palliative care unit)).

**Table 1.** Participant demographic data.

Geographic breakdown
Yukon: 3, British Columbia: 1
Alberta: 3
Manitoba: 5
Ontario: 2
Quebec: 5
Nova Scotia: 5
Profession
Social worker: 6
Physician: 6
Nurse: 8
Music therapist: 3
Occupational therapist: 1
Practice setting
Rural: 5
Rural/Urban: 1
Urban: 18
Religious and/or spiritual
Spiritual: 14
Religious: 1
Both: 7
Neither: 2
Racial identity
White: 23 (One of whom identifies as White/Francophone)
Asian: 1
Gender Identity
Male: 3
Female: 21
Age
30–39: 7
40–49: 7
50–59: 7
60+: 3
Years’ experience
3–9 years: 9
10–19 years: 14
20+ years: 1

### Data analysis

Semi-structured, 45–60 min telephone interviews were completed, audiotaped, and transcribed verbatim. Transcripts were the ‘texts’ that were analyzed for the presence of discourses.^66–68^ Asking questions of the data is a noted method within discourse analysis literature.^68–70^ The questions guiding data analysis for this study were informed by tenets of poststructural theory framing this study. The first question used to guide data analysis was: What patterns of topics are evident within and across the interview texts? Once patterns were identified, the second question was: What tensions or contradictions are evident within and across these topics? This is an essential, delimiting step in determining the presence of a discourse within the data, as not all topics identified contain tensions or contradictions. For example, participants described a tension in their day-to-day practice between the expectation to relieve patients’ nonphysical suffering and not necessarily being able to do so.. For example, one participant shared: ‘I think maybe that’s also—maybe that’s why it’s so difficult, in that you feel like you should be able to make a difference, but you can’t always’. The final question of the analysis was: What are the *effects* of the identified discourses? That is, what do the discourses *do*?^35,39,53,66,68,70–72^ As an example of a discursive effect, this same participant described feeling frustrated, sad, and inadequate when experiencing this tension in their work with patients. The data analysis was an iterative and interactive process, involving a continual movement between poststructural theory and the evolving understanding of the data. Insights emerged through sustained engagement with participants’ talk, guided by the analytic questions posed, and informed by theoretical commitments to discourse and its effects. Participants had the opportunity to review a research summary and their quotations to be used in the study write-up to ensure anonymity. Participants consistently shared, unprompted, how much study results resonated with their own experience, contributing to the study’s credibility.

### Researcher reflexivity

Both authors’ positionalities and entry points into this study share similarities and differences: both are female-identified social workers, educators, and scholars who aim to bring attention to diverse and less visible aspects of dying, death, and grief. At the time of the study, M.R. was engaged in doctoral studies and had 10 years of social work practice experience in a residential hospice setting. M.R.’s own struggle to relieve patients’ nonphysical suffering in this role informed the study’s conceptualization.^34^ M.R.’s background was not shared with participants (though some asked only after the interview’s end), and solely participants’ experiences were centered during data collection. The study was supervised by C.-A.C., a senior researcher, educator, and social worker with over 20 years of experience in grief and palliative care. M.R. conducted the interviews and completed the initial data analysis. Analysis was then verified and completed together with C.-A.C.’s analysis. The data analysis process involved regular, in-depth discussions, in which both researchers challenged each other’s ideas and assumptions about the data. Thick, descriptive extracts from the interview texts are intentionally included in this paper to enhance rigor and demonstrate how discourses within the data were constructed.^73–75^ The reporting of this study conforms to the COREQ checklist (Supplemental Material).^76^

## Results

The research team intentionally chose not to define ‘nonphysical suffering’ for participants. When asked at the interview’s start, ‘What words pop into your mind when you think of the nonphysical suffering you encounter in your work?’, participants consistently noted: ‘fear’, ‘anxiety’, ‘loneliness’, ‘alone’, ‘sadness’, ‘existential’, and ‘not wanting to die’. While the broader study identified four overarching discourses, this paper focuses on two interrelated and tension-filled discourses: (1) ‘Expectations of palliative care to relieve nonphysical suffering’ and (2) ‘Barriers to nonphysical suffering’s relief’ (see Table 2). Discourse 1 explores the multiple sources from which expectations to relieve nonphysical suffering arise, while Discourse 2 examines challenges to meeting these expectations—including the elusive nature of nonphysical suffering itself, and systemic, provider, and patient-level barriers. Additional discourses—on the personal impact of this work and on perspectives regarding medical assistance in dying—will be explored in future papers.

**Table 2.** Findings overview.

Discourse 1: Expectations of palliative care to relieve nonphysical suffering (i) Expectations from patients and families (ii) Expectations from medical teams outside of palliative care (iii) Expectations providers have of themselves Discourse 2: Barriers to nonphysical suffering’s relief (i) The nature of nonphysical suffering (ii) Systemic barriers (iii) Provider barriers (iv) Patient barriers

### Discourse 1: Expectations of palliative care to relieve nonphysical suffering

Across interviews, participants revealed a pervasive expectation—held by patients, families, and other medical teams outside of palliative care, and even palliative providers themselves—that palliative care should relieve nonphysical suffering. When this expectation cannot be met, it generates internal conflict, helplessness, and feelings of inadequacy among providers.

#### Expectations from patients and families

Participants described the emotional toll of facing patients’ and families’ expectations for relief from nonphysical suffering that cannot always be met. Two physicians captured this tension:

‘Why can’t palliative care make me feel better?’ and if you kind of point out that perhaps [nonphysical] issues x, y and z are contributing, that can be hard to hear. And, also if they’re contributing but you can’t fix it, then, I don’t know, they don’t want to accept that either.

. . .especially where some people might have been made to believe that palliative care is all about taking away the suffering. . . there’s the added burden of the family sort of looking at the provider, or looking at me, or I feel like they are looking at me, thinking or actually saying: ‘Isn’t there something else you can do? Look at my sister, can’t you see her suffering?’

#### Expectations from medical teams outside of palliative care

Colleagues outside palliative care were described as placing unrealistic expectations on the field, promising relief that providers know may be unattainable. One physician shared:

So often other services will sell palliative care—an enrolment in palliative care—by saying: ‘If you enroll on palliative care then you will have no pain’. And often when there’s a lot of nonphysical components to pain, having zero pain is not realistic.

A nurse participant echoed this idea:

I think sometimes they look to the palliative team as the one that’s going to solve everything, and you can’t always. And I guess for myself, I mean, I try my best to, to lessen it. I think I’m realistic in that I don’t think that in the last months of life or weeks of life that we are going to make a huge difference in people’s [nonphysical] suffering. I think we can do what we can for sure, work on the physical suffering to make sure that is done as much as possible.

#### Expectations providers have of themselves

Provider-held expectations were most prominently associated with feelings of inadequacy and helplessness. Participants struggled with the dissonance between their commitment to easing nonphysical suffering and the reality of its persistence. One physician reflected on an experience with a patient,

. . .who had very challenging pain symptoms, but we also thought that there was a layer of other suffering. She was a mother, there were a lot, just a lot of layers to her [illness] course. But, I just remember feeling inadequate and sad. Even though we don’t promise everyone, ‘We can make you comfortable’, we always are trying really hard, you know, for people to be as comfortable as possible, as peaceful as possible, and when a person isn’t, it’s hard not to feel like somehow I fell short.

A nurse participant similarly communicated:

I think maybe that’s also— maybe that’s why it’s so difficult, in that you feel like you should be able to make a difference, but you can’t always. And that’s frustrating because, well not frustrating, but it’s—and sad isn’t the right word either, only maybe it is the right word. . . We can try to maybe soften it a bit but, I don’t know. . .

Together, these experiences highlight the deeply felt sense of helplessness and inadequacy when providers are unable to meet internal and external expectations of nonphysical suffering’s relief.

### Discourse 2: Barriers to nonphysical suffering’s relief

A discourse about ‘Barriers to nonphysical suffering’s relief’ is visible within and across the interview texts. The nature of nonphysical suffering is itself identified as a barrier to its relief: how its hidden, less visible nature makes it difficult to identify and understand, and how the source of a patient’s nonphysical suffering may predate, and be amplified by, a life-limiting diagnosis. Multifaceted barriers at systemic, provider, and patient levels are also revealed.

#### The nature of nonphysical suffering

Nonphysical suffering was described as difficult to identify and understand due to its invisible, unspoken, and complex nature. Participants used terms such as ‘hidden’, ‘quiet’, and ‘what’s not being said’. An occupational therapist noted, ‘you really have to read between the lines’. And a physician shared: ‘I mean, I think the biggest challenge is identifying the non-physical suffering; being able to, (1) recognize that it’s there and, (2) being able to identify exactly what it is’.

Unlike physical pain, participants shared how nonphysical suffering resists categorization and demands interpretive effort. One nurse shared:

It’s just so different than physical [suffering]. . .it feels a lot easier to kind of categorize it when you’re looking [at it] physically. But, then, nonphysically it’s just a lot more, because it isn’t so visible, just all the extra work you have to do to really understand it.

Even when the word ‘suffering’ is explicitly used in patient encounters, another nurse shared how more time and effort are needed:

They could say, ‘I don’t want my dad to be in pain’. Well, then, you know exactly what they’re talking about. But as soon as they say, ‘I don’t want to suffer’, well then you have—there’s so much work to figure out what exactly are they meaning.

The study also found that a patient’s nonphysical suffering often predates their life-limiting diagnosis and is amplified by past trauma. One physician shared:

Often it’s not just at diagnosis and on the death bed that there’s psychosocial stressors. Often, it’s been accompanied for years and decades of life. And how do you help people through that? I don’t feel equipped, so I find that a bit overwhelming.

A nurse participant shared how past trauma may also be experienced at the community level, such as for Indigenous communities affected by colonial violence:

In terms of where we live, [a large percentage] of our population is First Nations and many went to residential schools and then. . . just, just that suffering involved with now people coming back into an institution[al setting], and then we have a lot of new staff not necessarily—there’s some—a lot of people come in from outside [this area] and so not having that understanding, and how that, just, it’s important to really recogniz[e] the source of someone’s suffering.

#### Systemic barriers

Participants highlighted how systemic constraints, particularly time pressures and limited resources, hinder their ability to address nonphysical suffering. The time required to engage meaningfully with such suffering is often unavailable in fast-paced medical settings.

A music therapist working in an inpatient palliative care unit shared,

I would say sometimes, just with the pressures with the medical system, sometimes it does get rushed or overlooked at times. . . some of the non-physical suffering actually takes a little bit more time to resolve, like it’s not as quick as a methadone rotation, you know? So, it’s been hard. Sometimes we’ve advocated just to keep somebody [on the unit] for their non-physical reasons, or for the emotional reasons, but, you know, it’s not always as popular.

Another social worker in inpatient and home care settings echoed this idea, noting that nonphysical suffering is ‘more tricky in lots of ways um ‘cause there isn’t, there isn’t as likely a quick fix, you know, or a medication. . . it’s not as, not as straightforward’.

Providers in both urban and rural settings also reflected on how these tensions are further exacerbated by the limited resources available within home care settings. One nurse working in a rural setting shared:

When I was in home care, if I had a patient who was an hour out of town, I know that I would be the only health care professional in there. So, I wanted to, you know, I had to quickly assess their needs and their—just try and address them in whatever capacity [I could]. So, yeah, I definitely felt, and you know, and the resource dry community, really there wasn’t a whole lot outside of my visit, or the visits of other nurses like me who could address them. So yeah, I felt pressure to try and relieve those things [like non-physical suffering].

While documented more in-depth elsewhere, the study also revealed that the absence, insufficient staffing, and/or underutilization of specialist psychosocial team members (e.g., social workers) on hospice and palliative care teams in both inpatient and home care settings creates barriers to patients’ nonphysical suffering being explored and supported.^77^

Some providers described the impact of resource constraints on themselves—an example of a discursive *effect*. A nurse participant working in palliative home care articulated the following:

The time we have, the resources we have, if those are inadequate, I find I take a lot of that on and I feel that deeply. . . I think the system wants to support people with wounds, so that’s very straightforward: you go in, you do wound care, you leave, you go to the next person. Or, they think it’s just these simple interventions. So, okay we have this person receiving palliative care at home; they need their medication drawn up and they need a Sub-Q line put in. But it’s not just that right? Like, I don’t think they understand the scope that’s required.

#### Provider barriers

Providers themselves face significant constraints in their work with patients’ nonphysical suffering. These include both late referrals to palliative care and rapidly changing patient conditions, wherein patients are too close to death to explore their nonphysical suffering. One physician working on an inpatient palliative care unit shared:

Sometimes we’re meeting people in the context of really advanced illness and they might be with us here for a day or two and then they’re gone. And how do you—how do you manage something so heavy when time isn’t on your side?

A social worker working in an inpatient hospice setting similarly noted,

A lot of the time the person is so sick already by the time I get there that I—I can’t possibly untangle what’s causing this [nonphysical suffering]. And so, it’s so hard to know how to—what to do in those situations.

Relatedly, participants described the need for palliative care providers to explore patients’ nonphysical suffering earlier in the illness trajectory.

Participants also described how they did not receive training specific to their work with patients’ nonphysical suffering. Many expressed feeling ill-equipped, uncertain about the scope of practice, or unprepared to engage with such nonphysical suffering, which is seen as more ambiguous and demanding than physical symptoms. A nurse working in home care shared that they find themselves,

. . .glossing over it because there’s not anything material that I can do about it, sometimes yeah, [it feels like I am] opening up a can of worms and, and not feeling like I have the skills or the tools to help them sort of ongoing with it, yeah I would say that’s definitely a challenge. . . it’s not something that we’re really trained to do, to deal with.

An occupational therapist also shared how there may be doubt about whether patients’ nonphysical suffering falls within their scope:

‘I don’t know if this is my scope of practice’, you know, because we are supposed to be working on function and independence and it’s like, but these things touch all aspects of someone’s life so of course it’s in our wheelhouse. So, I just see it as, like, just kind of a taboo thing that people think it’s not their—they don’t have the skills or they don’t have the permission to go there.

And a nurse participant spoke to their own preference to focus on more tangible aspects of care, and to what working with patients’ nonphysical needs may require from the provider:

You know it’s a lot easier for me I think to focus on things that I can fix, and it’s trickier, you know, for me to kind of personally open up and be available to kind of go into these nonphysical suffering type issues.

#### Patient barriers

Patient-level barriers are also evident. Participants noted that some patients struggle to articulate their nonphysical suffering. For example, a physician shared:

So I think part of what’s made it really challenging is that she’s not really been able to articulate any of that [nonphysical suffering]. Some people as you know are really able to put clear words to or even like [answer] to a direct question: What are you most afraid of? What concerns you most? They can sort of answer that, but she’s not someone who’s really ever been able to, she’s just—keeps using words like, ‘I’m just miserable’, and so yeah, that’s been part of the real challenge is not even knowing what, where, how do you approach? How do you reach towards someone who’s just in a place of misery?

Participants also spoke of patients masking their suffering out of politeness. An occupational therapist noted that patients may withhold sharing their nonphysical suffering with providers because they worry whether it is acceptable to do so:

[Patients] want to do for you what you want them to do. You know, they think they should get out of bed, they think they should mobilize, they think they should wear the stockings that you are providing them. But what’s not being said is, like, the fear that none of this is going to help, none of this is going to change the outcome, like, ‘Here I am, dying’.

Further speaking to how patients may hold back from sharing the whole of their nonphysical suffering with providers, a music therapist shared the following about working with a particular patient who was experiencing significant nonphysical suffering:

It’s tough to just like kind of go and like take it; you’re like, ‘I’m going to see him! Get ready!’ You know? And it was real, like very, very raw and very real. And I think that sometimes people are a little, like, polite about their emotional suffering. But he was just raw and honest.

Participants also spoke of patients who appear reluctant or resistant to express their nonphysical suffering, including resisting emotional engagement altogether. These dynamics often leave providers feeling frustrated or powerless in their efforts to offer support—both are examples of discursive *effects*. For example, one social worker shared,

When I have trouble is when, when I really feel like emotional expression would make this better and they can’t, or won’t. And not like in the short term; I feel I can be very respectful of that kind of stoic coping style and just let people be there, but it’s when there’s this, it’s when I sense this resistance. That’s what’s hard for me is resistance to being with what is. That’s what’s hard cause then there’s nowhere that I, that you, can move.

A music therapist also shared that some patients,

. . .will push everyone and everything away that might help crack it open so we can actually work with it, so they become kind of the most difficult for me personally. . . I find for me the most frustrating thing is when I don’t even get a chance, like, to me I feel like just, just give me one little chance to do what I think could help you.

## Discussion and implications

The two discourses—expectations for relief and barriers to its realization—operate in direct tension. While palliative care is discursively constructed as the space where suffering is alleviated, participants’ accounts reveal how nonphysical suffering eludes resolution due to multifaceted barriers. This leads to tensions in practice encounters with patients and families who expect its relief, as well as to provider feelings of helplessness, inadequacy, and frustration. This disjuncture offers a powerful site of analysis for understanding the limits and contradictions of palliative practice in relation to less visible forms of suffering.

### Increased understanding of multilevel barriers to nonphysical suffering’s relief

Study findings grow our understanding of barriers to the relief of nonphysical suffering at the patient, provider, and systemic levels. Barriers to nonphysical suffering’s relief have been only minimally discussed in existing research. Prior to the pilot study that preceded this study,^34^ the idea that nonphysical suffering is more difficult for providers to relieve than physical suffering had only been identified in one previous study of palliative care nurses.^19^ This finding was echoed in this present study as well. That nonphysical suffering cannot necessarily be medicated and does not have a ‘quick fix’—unlike physical suffering—was also noted in the 2017 pilot study.^34(p.676)^ These ideas—and exact words—were mirrored in this present study. The 2017 pilot study found that providers were *reluctant* to engage with patients’ nonphysical suffering due to the absence of a ‘quick fix’, time, and training.^34^ In contrast, this present study demonstrated that providers will *avoid* engaging with patients’ nonphysical suffering for these reasons. Past research has solely linked providers’ own feelings of discomfort to avoiding patients’ ‘unrelieved suffering’.^19^ Importantly, the present study is the first research to demonstrate that palliative care providers’ inability to relieve or ‘fix’ a patient’s nonphysical suffering can lead them to avoid engaging with patients’ nonphysical suffering—a key example of how discourses shape practice. Avoiding patients’ nonphysical suffering runs counter to the very aims of palliative care.

Study findings add to existing research that shows palliative care providers lack training specific to working with patients’ nonphysical suffering.^19,31,34^ Study results show this lack of training prevents them from exploring a patient’s nonphysical suffering; moreover, they may avoid asking about a patient’s nonphysical suffering because they lack tangible ways of relieving it. Notably, social workers and music therapists in this study identified patients’ nonphysical suffering as falling within their scope of practice,^77^ while other providers in the study (e.g., nurses, physicians, occupational therapists) questioned their role in relation to patients’ nonphysical suffering. While study findings indicate the need for more consistent and adequate staffing of specialist psychosocial team members,^77^ future research can explore each unique discipline’s training needs, as well as interdisciplinary training needs, specific to supporting patients’ nonphysical suffering. By understanding how discourses shape daily practice, educators and institutional policymakers can design more reflective training programs. Study results then contribute to the development of better support structures for palliative providers.

Prior research has shown that a lack of time and resources in medical systems precludes nonphysical suffering from being shared by patients or explored by providers.^13,78–82^ This present study’s findings help to expand this understanding by highlighting how nonphysical suffering is itself at odds with a fast-paced medical system. Nonphysical suffering is difficult to identify and understand because it is often hidden, less visible—its very nature requires more of providers’ time. Moreover, how the system is built for more ‘straightforward’ interventions, such as those focused on physical care needs and providing medication, is also revealed. In this way, one of many tensions palliative care providers navigate in their day-to-day work is revealed: nonphysical suffering requires significant provider time, and the fast-paced, resource-strapped medical system is time deficient. The medical system is not built for nonphysical suffering.

This is also the first study whose findings acknowledge the difficulty providers have both identifying and understanding patients’ nonphysical suffering. Existing palliative care literature and research focus primarily on ways of *relieving* patients’ nonphysical suffering, with meaning-making, cognitive restructuring, and fostering personal growth being long-standing, widely accepted responses.^9,13,83–88^ Less attention has been paid in the existing literature to helping interdisciplinary providers first *identify* and *understand* patients’ nonphysical suffering in their day-to-day practice encounters, prior to considering possible ways of bringing about its relief. This has significance for palliative care practice and training and represents a key area for further research.^13^

Study findings also reveal that patients present to palliative care teams very close to the end of life and/or their conditions quickly deteriorate, leaving insufficient time for nonphysical suffering to be addressed. This is another way in which this study revealed that providers lack time to respond to/support patients’ nonphysical suffering. Providers in this study further described feeling pressure to resolve patients’ nonphysical suffering before they die; this unique pressure of the ‘dying time’ within palliative care practice has not been previously captured. Participants suggested that a way to counter this pressure would be for providers to explore patients’ nonphysical suffering earlier in the illness trajectory; how interdisciplinary providers can be supported to specifically explore patients’ nonphysical suffering earlier in and along the illness trajectory warrants further research.

This study also revealed that patients’ nonphysical suffering may predate, and be amplified by, a life-limiting diagnosis—something that was first revealed in the 2017 pilot study.^34^ The present study provides additional insights around this: participants noted that patients’ preexisting psychosocial stressors, including trauma histories, may contribute to their experience of nonphysical suffering in the context of a life-limiting illness. Participants further shared how suffering of this nature may be particularly difficult to relieve by palliative care teams. Importantly, participants voiced how historical and ongoing colonialism and colonial trauma can be sources of nonphysical suffering for Indigenous Peoples. This suffering predates and becomes amplified in the context of a life-limiting diagnosis when accessing palliative care within medical systems that have historically been, and continue to be, sources of harm. The historical and ongoing harms of health care systems to Indigenous Peoples, and the need for culturally safe care, are well established.^88–91^ How these harms may impact patients’ experiences of nonphysical suffering is a vital consideration for teams providing palliative care to Indigenous communities and additional communities that disproportionately experience institutional harms, including Black, racialized, and 2SLGBTQ+ communities.^88–91^ Trauma-informed palliative care and practice have gained traction in recent years^92–94^; this present study adds to that body of literature by connecting palliative care patients’ trauma histories to their experiences of nonphysical suffering.

Another key finding is how patients’ care settings may impact whether their nonphysical suffering is sufficiently explored or addressed. Participants in both urban and rural settings consistently noted that patients receiving palliative home care had less access to support for their nonphysical suffering. This is a finding not previously identified in the existing literature and one that warrants further research—and funding. While access to palliative home care is itself limited in Canada,^95^ participants shared that palliative home care teams consistently lack specialist psychosocial care (e.g., social workers with specialist training in hospice and palliative care).^77^ Even inpatient settings were found to understaff and/or underutilize specialist psychosocial care, with consequences for patient care. More resource allocation, research—and advocacy—in this area is warranted.

That patients may struggle to articulate their nonphysical suffering to palliative care teams has been minimally recognized in existing literature.^7,81^ This aligns with Frank’s poignant description of suffering as ‘a lived reality that resists articulation’; it’s what your patients can’t say’.^96(p.353–354)^ Existing research notes that patients may underreport or minimize their nonphysical suffering so that they do not *themselves* become overwhelmed by it.^79–81^ This present study, however, found that patients may be ‘polite’ about their nonphysical suffering so that they do not overwhelm *providers* with it. That patients want to shield providers from the whole of their nonphysical suffering, to not burden them with it, is an important new finding. Moreover, that providers may perceive patients as ‘resistant’ to sharing their nonphysical suffering with them and feel frustrated when they are unable to ‘help’ was first noted in the 2017 pilot study^34^—these ideas were echoed in the present study. In direct tension with these findings, the study also revealed that providers find it difficult to bear patients’ nonphysical suffering when it *is* freely, openly, and rawly expressed. This patient/provider tension can prevent nonphysical suffering from being shared and potentially relieved. The above findings warrant further consideration and research into how they impact clinical care/practice.

### Tensions in practice

Study findings reveal, for the first time, a key tension and disconnect providers experience in their work with patients’ nonphysical suffering: the expectation that palliative care will be able to relieve patients’ nonphysical suffering, and its inability to necessarily do so. The strength of this expectation is another novel finding, stemming from patients and families, medical teams outside of palliative care, and within providers themselves. This tension/disconnect appears to be a source of ‘self-conflict’ for palliative care providers; that is, providers in the study describe a desire and pressure to relieve patients’ nonphysical suffering, while knowing this is not always possible. Notably, Dr. Eric Cassell described ‘self-conflict’ as a ‘constant feature of suffering’ for patients^20(p.274)^; that providers may too experience ‘self-conflict’ as a feature of their own suffering adds to existing understandings of ‘clinician suffering’ within palliative care.^13,33,97–100^ Social worker participants in the study spoke to a related tension in their practice: *knowing* they cannot always relieve a patient’s nonphysical suffering, while simultaneously feeling that they still *should* be able to, due to the psychosocial focus of their role.^77^

Notably, the two discourses outlined in this paper are in direct tension with each other, what O’Connor and Payne describe as ‘competing discourses’.^38(p.830)^ That is, the expectation that palliative care will relieve patients’ nonphysical suffering exists in opposition to the multilevel barriers providers encounter in trying to relieve patients’ nonphysical suffering. In their day-to-day practice, palliative care providers navigate discursive tensions and contradictions that affect them: study participants shared feeling helpless, inadequate, and frustrated in their work with patients’ nonphysical suffering. Their inability to relieve patients’ nonphysical suffering also resulted in them avoiding engaging with patients’ nonphysical suffering—the very antithesis of palliative care’s aim. Findings from four prior studies’ demonstrate provider feelings of helplessness and/or inadequacy in the face of nonphysical suffering that cannot be relieved^3,19,33,34^; in most of these study write-ups, such feelings are mentioned only briefly. White and colleagues’ study has been the only study, up until now, to hint at the link between providers’ inability to relieve nonphysical suffering, feeling helpless and inadequate, and the aims of palliative care; more than 20 years ago they wrote: ‘It is perhaps not surprising that nurses also reported a sense of failure when confronted with suffering that they could not relieve, given the underlying philosophy of palliative care’.^19(p.44)^ The present study’s findings importantly highlight the ongoing internal wrestling and struggle providers experience in their work with patients’ nonphysical suffering. The study’s poststructural discourse analysis methodology revealed these tensions and contradictions; using this theoretical and methodological framework in future palliative care research is warranted.

### Study limitations

The nearly all-white study sample must be acknowledged as not being neutral in its potential impact on the study results.^101^ There is a significant lack of racial diversity both in palliative care providers and in those accessing palliative care, and there is a connection between the two.^88–90,102^ In the context of this study, who accesses palliative care directly relates to whose nonphysical suffering is considered or captured by study participants.

As stated earlier, ‘nonphysical suffering’ is still not a concept commonly used in palliative care practice or research. It is possible that providers may have seen the study recruitment information and been unsure about the applicability of their experiences to the study criteria. While ‘spiritual care providers’ were specifically named on the recruitment poster as an example of the type of participant being sought (alongside ‘nurse’, ‘physician’, and ‘social worker’), ‘spiritual suffering’, specifically, was inadvertently omitted from recruitment material; the recruitment poster described nonphysical suffering as that which includes, but is not limited to psychological, emotional, social, and/or existential suffering. While spiritual care providers work with and support all kinds of suffering, this omission may have dissuaded some from participating. As a key member of the palliative care team that regularly engages with patients’ nonphysical suffering, the absence of spiritual care providers in this study represents a gap; encouraging their participation in future research focused on patients’ nonphysical suffering is imperative.

## Conclusion

Since the late 1960s, hospice and palliative care literature and discourse have long ascribed the relief of nonphysical suffering to a patient’s ability to transcend their own suffering, and to a palliative care provider’s ability to facilitate or support that transcendence. The present study’s findings call into question this long-standing discourse and expand our understanding of nonphysical suffering by urging the field to consider how multiple intersecting barriers at patient, provider, and systemic levels shape—and limit—palliative care’s aim to relieve nonphysical suffering. The difficulty of identifying and understanding nonphysical suffering, as well as the fact that it may predate and be amplified by a life-limiting diagnosis, are additional ways this research deepens our understanding of nonphysical suffering and the barriers to its relief. In these ways, this study reveals a significant daily practice tension not commonly addressed in palliative care literature and research: the expectation that palliative care will relieve patients’ nonphysical suffering, its inability to necessarily do so, and the resulting impact on providers and the patient–provider encounter.

## Supplemental Material

sj-pdf-1-pcr-10.1177_26323524251379910 – Supplemental material for ‘It’s hard not to feel like somehow I fell short’: A discourse analysis of palliative care providers’ experiences with patients’ nonphysical suffering Supplemental material, sj-pdf-1-pcr-10.1177_26323524251379910 for ‘It’s hard not to feel like somehow I fell short’: A discourse analysis of palliative care providers’ experiences with patients’ nonphysical suffering by Maxxine Rattner and Cheryl-Anne Cait in Palliative Care and Social Practice
